# Paradoxes in the Ontological Classification of Glia—Evidence for an Important New Class of Brain Cells with Primary Functions in Iron Regulation

**DOI:** 10.3390/cells15060511

**Published:** 2026-03-13

**Authors:** Adrienne E. Milward, Rebecca J. Hood, Chan-An Lin, Conceição Bettencourt, Elvis Acquah, Jake Brooks, Joanna F. Collingwood, Yoshiteru Kagawa, Samantha J. Richardson, Yuting Wu, Yi Lu, Mirella Dottori, Daniel M. Johnstone

**Affiliations:** 1 Faculty of Science, Medicine and Health, School of Medical, Health and Indigenous Sciences, University of Wollongong, Wollongong, NSW 2522, Australia; cal875@uowmail.edu.au (C.-A.L.); mdottori@uow.edu.au (M.D.); 2 The Florey Institute, University of Melbourne, Parkville, VIC 3052, Australia; yoshiteru.kagawa@florey.edu.au; 3 Faculty of Health and Medical Sciences, School of Biomedicine, University of Adelaide, Adelaide, SA 5005, Australia; rebecca.hood@adelaide.edu.au; 4 School of Biomedical Sciences and Pharmacy, College of Health, Medicine and Wellbeing, The University of Newcastle, Callaghan, NSW 2308, Australia; daniel.johnstone@newcastle.edu.au; 5 Department of Neurodegenerative Disease, Queen Square Institute of Neurology, University College London, London WC1N 3BG, UK; c.bettencourt@ucl.ac.uk; 6 Department of Pathology, ARUP Institute of Clinical & Experimental Pathology, School of Medicine, University of Utah, Salt Lake City, UT 84108, USA; elvis.acquah@hsc.utah.edu; 7 The Biomedical Engineering Institute, School of Engineering, Faculty of Science, Engineering and Medicine, University of Warwick, Coventry, CV4 7AL, UK; jake.brooks@warwick.ac.uk (J.B.); j.f.collingwood@warwick.ac.uk (J.F.C.); 8 School of Science, STEM College, RMIT University, Melbourne, VIC 3000, Australia; samantha.richardson@rmit.edu.au; 9 Department of Chemistry, The University of Texas at Austin, Austin, TX 78712, USA; yuting.wu@austin.utexas.edu (Y.W.); yi.lu@utexas.edu (Y.L.)

**Keywords:** Iron, glia, oligodendrocyte, myelin, evolution, mental health, Alzheimer’s disease, stroke, cancer, DNAzyme sensors

## Abstract

The ontological categorization of the cellular elements of the brain was proposed over a century ago by Santiago Ramón y Cajal (neurons, astroglia) and Pío del Río-Hortega (oligodendroglia, microglia). It combines histochemical observations of morphology with allied inferences about the specialized functions and origins (ectoderm or mesoderm) of each cellular element. This ontology shapes modern neuroscience, with the main non-neuronal cells—astroglia, oligodendroglia and microglia—viewed as having distinct primary roles relating respectively to the metabolic support, myelination and immunoprotection of neurons, the information signaling cells. Yet contemporary techniques, ranging from electrophysiology to single-cell transcriptomics and ultrahigh resolution spectroscopy, are revealing intersecting molecular profiles and functional capacities of these cell groups, for example metabolic support, neuroimmune and signaling functions in oligodendroglia. Here we identify discrepancies in current glial paradigms, from empirical, evolutionary and pragmatic perspectives. We suggest a subset of small, iron-rich glial cells, usually with few processes, often viewed as oligodendroglia with myelin-related primary functions, instead have iron-related primary functions that are central to all aspects of brain activity. We call these ‘ferriglia’. We discuss implications for pathogenesis across the spectrum of neuropsychiatric and neurological disorders, including neurodegenerative conditions such as Alzheimer’s disease and other less common cognitive, movement and neurobehavioral disorders, stroke and cerebrovascular disease, glioblastoma and other brain cancers and neuroimmune conditions. We also briefly address the question of where ferriglia may reside within existing glial compartments and lineages, implications for the ontological classification of other glial cells, and research challenges that must be overcome going forward.

## 1. Introduction

“I am a firm believer that, without speculation there is no good & original observation.”

Charles Darwin, letter to Alfred Wallace [1,2].

The term ontology is often used in biology to describe a set of concepts and categories that aims to facilitate the understanding of a subject area or knowledge domain [3,4]. The accepted ontological categorization of the main cellular elements of the central nervous system (CNS) as neurons, macroglia (astrocytes, oligodendrocytes) and microglia arose from histochemical studies over a century ago [5,6,7,8,9,10,11,12,13,14,15]. The primary function of each element was inferred from circumstantial structural and contextual evidence gleaned from histological observations. Information exchange by neurons was deduced in part from their interconnected dendritic networks, metabolic support by astrocytes from their propensity to form bridges between neurons and vessels, immune responses by microglia from their clustering around pathological lesions and electrical insulation of neuronal axons (‘myelination’) by oligodendrocytes from the wrapping of their processes around axons.

We are entering a new era of understanding as modern biotechnologies such as novel chimerization models, single molecule arrays and multiomics profiling of individual CNS cells [16] provide new feature sets for increasingly precise ontological classification. This has led to proposals for new subgroupings within each of the astroglial (e.g., [17,18,19]), microglial (e.g., [20,21,22]) and oligodendroglial (e.g., [23,24]) classes.

As many as nine or more different subtypes of astroglia may exist, distinguished by spatial localization, molecular profiles and responses to inflammatory stimuli that may differ between species, sexes, brain regions and health or disease states [17]. Different subtypes of oligodendroglia (OLG) have also been proposed, that may likewise vary by molecular profile, spatial localization or other factors [24,25,26,27,28,29,30]. It is unclear how, if at all, such astroglial and oligodendroglial phenotypes reflect reactive, as opposed to inherent, phenotypes. Similarly, there may also be multiple phenotypically distinct microglial subtypes with distinct properties and functions that respond differently to stimuli, including so-called rod microglia and ‘dark microglia’ [20,21,22,31,32]. Some microglia subgroups are proposed to have different inherent phenotypes and functionalities, rather than being functionally different facets of a single inherent highly plastic phenotype that alters in response to environmental factors, as once believed [20,21,22].

These new findings entail fundamental departures from past views of glia cell groups. For all glial classification systems, a crucial distinction needs to be made between subtype classification based on innate features that reflect inherent subtypes, as opposed to plasticity reflecting reactive states [21,33,34]. It is important to determine whether different astroglia, microglia or OLG subgroups observed in different brain regions or in studies with different conditions are indeed innately different subtypes, rather than different reactive states.

Separation between different cell types of essential inherent primary functions that may otherwise come into conflict is likely to have evolutionary advantages. For example, having one group of cells with the primary function of migratory defensive responses to life-threatening pathogens and another group with the primary function of delivering essential nutrients to neurons helps prevent cells becoming trapped in a stasis of mutually exclusive, conflicting responses.

Conversely, there are likely evolutionary benefits in redundancy of vital functions across different glial types. The primary, definitive, functional feature of microglia has been their neuroimmune responses, including phagocytosis. Yet CNS defense is now also considered an important function of astroglia, supplementing their recognized roles in CNS metabolic homeostasis with many immune response functions shared with microglia, including phagocytosis, and with overlapping targets and molecular profiles [13,35,36]. These redundant functions may expand or possibly even override primary astroglial functions in pathological or other unusual circumstances where microglia responses are inadequate. Similarly, OLG can exhibit metabolic or neuroimmune functions, in addition to primary functions related to myelination [37,38,39,40,41].

It is unknown if function-based classification alone can distinguish glial species effectively without also considering other features that may not necessarily have clear involvement in vital activities e.g., morphology or structural markers such as glial fibrillary acidic protein (GFAP). Increasingly precise subclassifications, functional or otherwise, are of limited use, and may even be invalid, if the ontology itself is not an accurate representation of the biological entities being classified. For example, consider two cell groups arising from a common ancestor during evolution that have since diverged and perform very different, possibly even mutually exclusive, functions but retain some shared phenotypic features. Classifications focusing on shared phenotypic features, blind to divergent primary functions, will not usefully distinguish subgroups. We believe there are important blind spots of this kind in the present glial ontology.

Here we challenge the accuracy of the existing ontology and suggest some cells in recognized glial categories have essential primary functions beyond the triad of immune response, general metabolic support and production of myelin, the electrical insulating material ensheathing some axons. This is exemplified by the OLG, even though this lineage might perhaps appear to have the clearest grounds for function-based classification of the three main glial types. Oligodendrocytes (myelination-competent cells) and their committed progenitors have near unique, non-redundant, direct or indirect functions in myelin production that do not appear to be readily subsumed by cells considered to be microglia or astroglia, except in artificially engineered scenarios [42,43].

Yet despite this apparently unequivocal function, we propose some cells often viewed as OLG instead have essential primary functions relating to maintenance of brain iron homeostasis, that are independent of myelin-related functions. We suggest these cells are able to load, store and transport relatively large amounts of iron in healthy brain and often exhibit strong labeling for iron by 3,3′-diaminobenzidine-enhanced Perls’ stain (DAB+Perls’) for ferric (Fe^3+^) iron [44]. It is not clear whether these cells reside in existing glial compartments and lineages or instead have separate, unrecognized compartments, lineage/s or both. Consequently, we call these cells ferriglia (Fe+glia) to distinguish them from other glia (notably astrocytes, microglia) not specialized for iron handling, which may acquire iron in some pathological circumstances but do not usually accumulate comparable amounts of iron in healthy brain.

**Figure 1:**
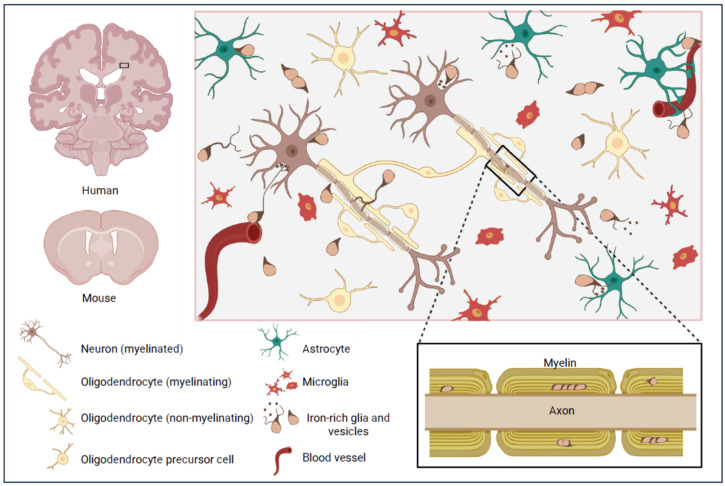
**Promiscuous relationships of iron-rich glia that may masquerade as OLG.** While glia sometimes exhibit distinctive morphologies, inferring species or functions from location and morphology alone can be unreliable. This is exemplified by small iron-rich glial cells (‘Fe+glia’), which usually have few processes and eccentric nuclei, features often ascribed to OLG, but may instead be an unrecognized cell group. Some Fe+glia engage in proliferative activities in iron-rich neighborhoods, including myelin-ensheathed fiber tracts, or nestle beside vessels, neurons or other cells [57]. Fe+glia may participate in iron-transfer mechanisms via iron-laden extensions, possibly involving transferrin, clathrin-mediated endocytosis or both or ‘kiss and run’ or other exchanges of iron-containing vesicles [58,59,60,61]. Figure created using BioRender.com.

**Figure 2:**
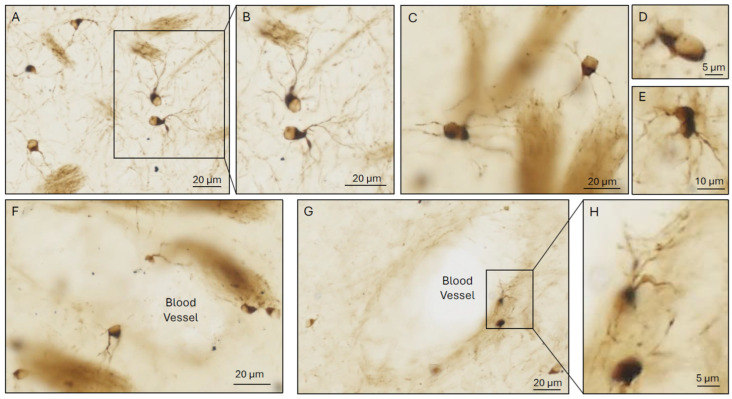
**Iron-rich glia (Fe+glia) in wildtype AKR/J mouse brain at age 9 months**. Small cells, often of diameter <10 μm, with eccentric nuclei and few processes (i.e., morphological features often attributed to OLG), frequently show intense cytoplasmic labeling with DAB+Perls’ stain for iron [44], with little nuclear labeling. Photomicrography (Axioscan, ZEISS, Jena, Germany) shows fine plexuses of criss-crossing cell processes (e.g., **A**,**B**,**G**), as described by Hortega for OLG (Section 3.3.2). Interstitial Fe+glia (**A**–**C**,**F**) also often make apparent contact with iron-rich, fibrous, myelin-like structures some distance away via very long fine processes that appear poorly suited for stable myelination. These may instead be transient extensions for uni- or bi-directional transfer of iron or other substances between myelinated reservoirs and neurons or other cells. Some Fe+glia display mitotic figures consistent with proliferation (**D**,**E**). Juxta-vascular Fe+glia are also seen (**F**–**H**), sometimes adjoining iron-rich myelin-containing structures (**F**). These occasionally appear to extend fine processes into the vessel lumen, consistent with uni- or bi-directional transfer of iron or other substances to or from blood (**G**,**H**). (Milward et al., unpublished images).

Specific protein markers for Fe+glia are needed. Currently, our main criterion for distinguishing these cells from other glia is histochemically detectable ferric iron, with little if any expression of markers for glial subclasses such as astrocytes, microglia, pericytes or OLG. Other common, but not essential, features are small size, eccentric nuclei, oval or raindrop shape and few primary processes (Figure 1 and Figure 2; Section 3.1.2 and Section 3.4). Fe+glia may also display mitotic figures indicative of proliferation (Figure 2; Section 3.3.4). We hypothesize Fe+glia provide a safe and efficient ‘iron supply chain’ within the brain that confers powerful survival advantages, protecting against iron toxicity or iron-scavenging pathogens. From evolutionary and pragmatic perspectives, essential primary roles in iron homeostasis such as transferring iron between blood vessels, neurons, astrocytes and other glia, and myelin, are unlikely to be compatible with competing primary roles in myelination that tether cells to myelin for long periods. We believe these considerations necessitate reassessment of the validity and utility of classical OLG ontology, and deeper contemplation of how this might require rethinking ontological classification of CNS cells in general.

## 2. History

The non-neuronal component of the nervous system was first described by 18th century European scientists and named neuroglia or simply ‘glia’, from the Greek word for glue, by Rudolph Virchow in 1856 [31]. This reflected prevailing beliefs that ‘glia’ consisted of a connective structure binding together the neural tissue [12,31,45], though other scientists such as Carl Bergmann contemporaneously published descriptions of non-neuronal cellular entities, reviewed [46]. We use ‘neural’ to encompass nervous system components in general, including glia, and ‘neuronal’ specifically for neurons. Virchow also coined the term ‘myelin’ and proposed that myelin provide electrical insulation for axons [47].

### 2.1. Cajal’s Classification of Neurons and Astroglia

The first systematic ontology of CNS cells, and forerunner of the current ontology, is credited to Santiago Ramón y Cajal [6,7,45,48], and included the concept of neuroglial cells. Cajal proposed three anatomically and functionally distinct groups of CNS cellular entities that he termed elements [8,9,14,48]. He hypothesized that information-related nervous system functions are performed by cells we now call neurons, which he categorized as the first cellular element of the CNS. In the early 20th century, the non-neuronal ‘glue’ component of the CNS was still considered by some prominent early histologists, including Camillo Golgi, to be an extended, interconnected, reticular network or syncytium [49]. Cajal instead proposed that star-like cellular entities (astrocytes) comprised a second ‘neuroglial’ element providing nutritional and metabolic support to neurons [9,50,51].

### 2.2. Hortega’s Classification of Microglia and Oligodendroglia

Cajal incorrectly proposed a third CNS element of small, apolar cells without detectable processes by Golgi staining [8]. Pío del Río Hortega developed an improved silver carbonate stain [52] revealing fine ‘elongations’ (appendages or processes) and proposed the present classification of glia based on phenotypic features, postulated embryonic source (ectoderm or mesoderm), location and inferences about function [41,53]. Some cells described by Hortega, which he named microglia, for their small size, were commonly seen in gray matter [54,55]. He proposed microglia were the true ‘third element’, originating from the mesoderm outside the CNS, unlike the first two elements, both of ectodermal neuroepithelial origin, neurons and macroglia, which at the time comprised protoplasmic and fibrous astroglia [41,54,55,56]. Hortega correctly deduced migratory, surveillance and phagocytic functions of microglia from histochemical observations across varied physiological and pathological contexts [11,14]. His classification of microglia as the third element was thus multifaceted, based on inferences about their non-neuroglial nature (originating outside the CNS), as well as their morphology (small size, amoeboid or ramified shape), functions and greater density in gray compared to white matter.

Together, these features distinguished microglia from OLG (see Introduction), the other new cell group defined by Hortega [10,11,12,14,41,53,54]. Hortega’s descriptions of OLG initially focused on apolar ‘interfascicular’ cells grouped in bundles or columns between nerve fibers in white matter, then expanded to a wider range of morphologies and locations, including gray matter (Section 3; [10,11,12,14,41,53]). He later named these cells ‘oligodendroglia’, from Greek terms signifying scarce or few (‘oligo’) and processes (‘dendro’).

Hortega proposed oligodendrocytes were the myelinating cells of the brain, describing OLG with fine processes spiraling around myelinated nerve fibers [10]. Hortega’s findings were validated by Wilder Penfield in 2024 [62]. Examples of OLG and other glial morphologies are depicted in Figure 1. Hortega categorized OLG as macroglia, i.e., ‘large glia’, the second element that already contained the astroglia, thereby establishing the accepted ontological classification of the three major recognized glial species that prevails a century later [8,10,11,12,13,14,15,16,31,41,46,53,54,55]. The second macroglial element also came to include other glia such as ependymal cells, recognized as being cellular in nature in the late 1800s [63,64], before Cajal’s classification.

## 3. Paradoxical Properties of Cells Currently Classified as OLG

The cellular ontology of CNS neurons and glia developed by Cajal, Hortega and other pioneering histopathologists was an extraordinary achievement, given the complexity of the brain, the paucity of foundational knowledge and technical constraints on the features able to be interrogated at the time. Yet even in Hortega’s original descriptions, the cell group classified as OLG was recognized to have paradoxical properties not inevitably consistent with primary functions in myelination [10,41,53]. Hortega studied OLG in diverse regions of brain or spinal cord from several vertebrate species (e.g., monkey, newborn or adult human), under physiological or pathological conditions. Over time, as his observations expanded across different physiological and pathological contexts, his perceptions of what constituted and defined OLG as separate from other elements also shifted.

### 3.1. Invalid Presumptions Arising from Inferring Myelination or OLG Identity from Cell Size

Many early histologists, including Hortega, already recognized the problems of categorizing glial types by morphology alone. Morphology is plastic and changes with many factors, including cell cycle phase and interactions with other cells, activation state, aging, nutrients, toxins and other environmental variables. Yet it is still widely used, either alone or in conjunction with location, to identify OLG, unsupported by cell marker labeling. Here we consider anomalies relating to OLG morphology.

#### 3.1.1. Size

One perplexing discrepancy in descriptions of OLG by Hortega and later researchers involves their size. While usually grouped with astroglia as macroglia, rather than ‘microglia’, some OLG are depicted as considerably smaller than astroglia by Hortega (reproduced [14]) and others [65]. Hortega himself noted many cells he considered to be OLG were similar in size to microglia and easily confused without staining of cell processes: …‘as the microglial elements, and many of the oligodendroglia are small, and all of them with prolongations, confusion is easy when dealing with rounded nucleus cells and incompletely stained protoplasmic appendages and without their real features’… (translated Iglesias-Rozas and Garrosa [66]). In this passage, Hortega considered OLG to be distinguished by their close relationship with nerve fibers yet he elsewhere described cells he considered to be OLG in interstitial or juxta-vascular locations without clear relationships with nerve fibers. (Juxta-signifies ‘near or adjacent to’ and peri- signifies ‘surrounding or around’ e.g., juxta-vascular OLG; peri-axonal myelin sheath.)

#### 3.1.2. Other Morphological Features—Eccentric Nucleus, Shape and Fine, Sparse Processes

In Hortega’s diagrams, some OLG have a central nucleus and processes spiraling round multiple axons, yet other OLG have an eccentric nucleus and, consistent with their name, few (‘oligo’) processes (‘dendro’) [10,14,41,53], illustrated in Figure 1 and Figure 2. Glia with similar oval or ‘raindrop’ shaped morphologies, with eccentric nuclei and only a few processes, are still often assumed to be OLG, even without marker studies or other evidence.

These anomalies have persisted in later research, which still widely assumes myelination is the primary function of cells with this distinctive morphology, often inferring OLG identity and myelinating capabilities from morphology alone, without marker validation. Yet, as discussed below, this group of glia is recognized to sometimes exhibit paradoxical properties not clearly consistent with primary myelinating functions, including locations not near myelin [10,41,53,67,68] and mitotic figures [69].

### 3.2. Invalid Presumptions Arising from Inferring Myelination or OLG Identity from Location

Although Hortega distinguished OLG from microglia as being more common in white matter, he also described abundant OLG in gray matter. Besides four myelinating oligodendrocyte subtypes [41,53], he also described cells with OLG-like morphologies in apparent non-myelinating contexts in juxta-vascular, juxta-neuronal and interstitial locations in white and gray matter. Though electron microscopy shows some juxta-neuronal OLG myelinate neuronal cell bodies [70], other OLG-like cells appear to be neither myelination-competent oligodendrocytes nor committed progenitors [71].

#### 3.2.1. Nature and Roles of Myelin-like Structures at Juxta-Neuronal or Other Non-Axonal Sites

Even when OLG are engaged in peri-neuronal ‘myelination’ at non-axonal sites, this ‘myelin’ may differ in wrapping structure or other salient features from peri-axonal myelin. As described in Section 5.5.1 below, the latter serves to accelerate electrical signaling via saltatory conduction and confer energy savings [72,73], in addition to providing metabolic support [37,38], for example nutrient delivery or waste clearance, and protecting nerve fibers from chemical or physical damage. Myelin-like lipid-rich substances that are not wrapped around axons may also provide physical protection or metabolic support to the neuronal cell body yet may differ in functions or effects on neurotransmission, compared to axonal myelin. For example, nutrients required by the soma may differ from those along long axons. This is relevant when considering the relative importance and competing requirements of myelination versus other vital functions in which OLG or OLG-like cells might be engaged, such as transporting iron or other essential nutrients, or waste, between blood vessels, brain cells and iron storage reservoirs. Notably, we propose the brain may also contain myelin-like structures which may act as iron storage reservoirs and are not apposed to or wrapping any neuronal components (e.g., see Section 5.1.2).

#### 3.2.2. Assumptions About the Nature and Roles of OLG-like Cells at Juxta-Myelin Sites

Conversely, even OLG near myelin may not have primary myelin-related functions, as noted by Wood and Bunge: …‘demonstration of the connections between oligodendrocyte somas and myelin sheaths is inherently very difficult, however, and it is possible that there are substantial numbers of cells in white matter, resident among the myelinating oligodendrocytes, that do not directly husband myelin segments’… [71].

We note that even processes clearly extending to myelin sheaths may not be myelinating. Fine processes from OLG-like cells to myelinated axons across interstitial expanses several times the length of the soma are depicted by Hortega [10,11,12,14,41,53,56] and others [65] in classical diagrams. These appear to be an inefficient and vulnerable strategy for long-term myelination of axons, but a feasible, readily relocatable, ‘pop-up’ pipeline for transfer of iron or other materials between myelin or other myelin-like iron storage sites and blood vessels, neurons or other cells. Figure 2 shows iron-rich cells (Fe+glia) with OLG-like morphologies extending iron-rich processes across interstitial expanses to myelin-like structures (Figure 2A–C,F) or into vessels (F–H) or undergoing mitosis (C–E).

### 3.3. Non-Myelin Related Functions of Subsets of Cells Usually Assumed to Be OLG

It is often assumed non-myelinating cells with OLG-type morphologies described by Hortega and others [10,11] are quiescent oligodendrocytes that can myelinate in appropriate circumstances [71,74] or oligodendrocytes with other roles supplementing primary functions related to myelination [71,74,75,76,77]. This is largely unexplored, perhaps because such roles are usually deemed subsidiary to the primary function of myelination. Below we consider features of OLG and related cells in juxta-neuronal, juxta-vascular or interstitial locations, focusing on criteria used to identify these cells as OLG and proposed non-myelin related functions.

#### 3.3.1. Juxta-Neuronal OLG

Juxta-neuronal OLG, i.e., near the neuronal cell body—perikaryon—as opposed to the axon, are also called perineuronal or satellite oligodendrocytes. These cells are often immunoreactive for 2′,3′-cyclic nucleotide-3′phosphodiesterase (CNPase) and are found throughout cerebral gray matter, at greater density in deep cortical compared to superficial layers [77,78]. The CNPase marker labels oligodendrocyte precursor cells (OPC) and committed oligodendrocyte precursors (COP), as well as mature oligodendrocytes, and may be a marker for commitment to eventual myelin formation [79], although CNPase labeling does not necessarily imply myelination.

The literature includes reports of both myelinating and non-myelinating juxta-neuronal OLG. Electrophysiology, electron microscopy and other approaches show that some juxta-neuronal oligodendrocytes in layer 5 of mouse neocortex myelinate surrounding axons [70]. This study found these oligodendrocytes also have other functions and can integrate with astrocytes in a glial syncytium that buffers K^+^ and constrains high-frequency neuronal action potential firing. This revitalizes early concepts of glial reticular networks championed by Golgi and others [49] (Section 2.1 and Section 3.3.2), equivalent to ‘functional syncytia’, and suggests descriptions of astroglial networks (e.g., [80,81]) should incorporate details of interconnections with OLG.

In contrast, another study examining cell markers and gene/protein expression profiles concluded some juxta-neuronal OLG are non-myelinating cells with a unique phenotype not consistent with other known OLG subtypes, expressing oligodendrocyte transcription factor 2 (OLIG2) but also other transcription factors and platelet-derived growth factor (PDGF) receptor-αβ and its ligand PDGF-CC [82].

Whether myelinating or not, juxta-neuronal oligodendrocytes may provide metabolic and functional support to neurons. For example, glutamine synthetase produced by some juxta-neuronal OLG is needed for normal glutamatergic neuronal transmission of nearby neurons, independent of myelinating functions of these OLG [83,84]. Changes in juxta-neuronal oligodendrocyte numbers, distribution or both are also observed in a number of neurological disorders [78]. It is unclear if there are distinct subsets of juxta-neuronal OLG with different inherent properties or if observed differences in health or disease are instead plastic responses to environmental factors, analogous to the debate on microglia mentioned above [85].

Research is also needed on whether juxta-neuronal OLG or OLG-like cells supply iron or other metals to neurons in healthy brain, whether this may be compromised in some disease states and potential translational implications.

#### 3.3.2. Juxta-Vascular OLG

Relationships of OLG with brain vasculature are unclear. Some OPC may use vessels as a scaffold for migration, detaching when differentiating into mature oligodendrocytes [86], but mature oligodendrocytes may also associate with vessels. Hortega detailed the complex expansions of plexuses formed by OLG (reproduced [10,11,12,14,41,53,56]), including apparent non-myelinating juxta-vascular plexuses of ‘criss-crossing’ OLG processes. (Criss-crossing plexuses of Fe+glia processes can be seen in Figure 2) Hortega acknowledged the plexuses he observed could be interpreted as neuroglial reticular systems, similar to extended single entities with fused cytoplasm proposed by pioneering pathologists such as Virchow and Golgi [49], early versions of modern ‘functional syncytia’ (Section 3.3.1). Yet he himself believed such fusion does not occur and the cytoplasm of individual OLG does not anastomose with that of other OLG, astrocytes or microglia in a single, interconnected structure.

This area is rarely studied, but comprehensive examination of OLG in brains from young adult mice (age 6–12 weeks) estimated that about one in six CNPase-immunoreactive OLG are in close proximity (<1 μm) to the brain vasculature, in particular capillaries. Plexus formation was not mentioned, but while the nature of functional interactions between OLG and vessels remains unclear, there was evidence of direct contact with the vascular basement membrane, suggesting exchange of information (signaling networks) or metabolites with endothelial cells [86]. Similar considerations may apply to iron-rich, OLG-like cells (Fe+glia) in close proximity to vessels, conceptually depicted in Figure 1.

Relationships between juxta-vascular OLG and iron regulation are poorly understood. Research in human brain by the Connor and Moos groups, Morris and others has shown that juxta-vascular cells with morphologies similar to OLG contain radiochemically and histochemically detectable iron and iron-related proteins [67,87,88]. This is consistent with roles in iron transport and uptake from endothelial cells into the brain although these functions remain to be confirmed.

Figure 2 shows complex nets of fine, iron-rich Fe+glial processes near vessels and in interstitial spaces, that appear more consistent with primary roles in iron transfer than in myelination.

#### 3.3.3. Interstitial OLG

Cells with OLG-like morphologies have been observed scattered throughout the brain in humans, primate, rodents and other mammals such as sheep [89], singly or in doublets (including mitotic figures) or small clusters, without apparent contact with myelin, neurons or vessels [67,68,69,76,90,91]. The functions of these cells are unknown but may include immune responses with secretion or uptake of cytokines and chemokines [37,39,40]. The observations that primary OPC exhibit phagocytic capabilities and, when exposed to IFNγ, can cross-present antigens to cytotoxic CD8 T cells in vitro also demonstrate immune-like functions of OLG cells [92,93]. Such findings suggest that cells currently considered to be OLG can adopt an altered phenotype resembling that of immune cells under both normal and disease conditions. Again, it remains to be seen whether such OLG have different inherent properties or if observed differences in health or disease are instead plastic changes, as discussed above.

#### 3.3.4. Progenitor Cells of the OLG Lineage

Observations of mitotic figures in some cells with OLG-like morphology in juxta-axonal and other locations are not consistent with these cells being oligodendrocytes, which are post-mitotic [94,95]. Such cells are instead often assumed to be OPC. As mitotic figures often occur in Fe+glia with OLG-like morphologies, these cells could also be OPC. Yet OLG precursor/progenitor cells labeled by markers such as nerve/glial antigen 2 (NG2; also known as chondroitin sulfate proteoglycan 4) or PDGFR-α [96,97,98] typically display very different morphologies. These appear considerably larger than Fe+glia and, to our knowledge, there has not been clear demonstration of ferric iron in cells co-labeled for these markers.

#### 3.3.5. Olfactory Ensheathing Glia

Olfactory information is sent from peripheral sensory neurons in olfactory epithelium to terminal synapses with neurons in the olfactory glomeruli. The olfactory nerve has non-myelinated axons, surrounded by a special type of glia, olfactory ensheathing cells (OEC), with features of both astrocytes and Schwann cells. These cells support neural transmission and olfactory sensory neuronal regeneration and participate in innate immune responses [99,100,101], suggesting considerable functional diversity.

The OEC can myelinate in some experimental settings and express CNPase [79]. Based on MRI and ferritin levels, OECs develop high levels of iron at the glia limitans (between the cerebral cortex and the pia mater) in the olfactory bulb by age 12 weeks in wildtype mice [102] and so may constitute a form of Fe+glia. Olfactory lobes arose early in brain evolution. The observation of early iron-rich ensheathing cells that do not myelinate under normal conditions is consistent with these cells having other functions and possibly also (or instead) with ensheathement serving other functions that preceded the subsequent rapid saltatory conduction function of myelin, as discussed in more detail below (Section 5.3.1, Section 5.4 and Section 5.5).

The olfactory bulb also contains oligodendrocytes or other OLG [101,103], thought to be generated locally from progenitors that may express proteolipid protein (PLP) but not platelet derived growth factor receptor (PDGFR) [104]. These OLG interact with OEC and may myelinate dendritic segments and neuron bodies surrounding olfactory glomeruli [101,103]. There is little information on their iron content and relationships to OEC, if any.

### 3.4. Limitations of Past Research

When exploring non-myelin related OLG features to identify novel functions, as above, it is important to distinguish changes in cell species or type (i.e., the inherent identity of the cell) from plastic changes in cell state, sometimes termed reactivity—this can have functional or clinical implications [20,21,105]. For example, functions involving neuroimmune factors such as cytokines or chemokines might fluctuate reversibly in OLG in responses to stressors while the broader phenotype that defines cell type remains unchanged.

Another limitation is that researchers usually seek non-canonical OLG functions by *first* selecting for OLG using markers specific for oligodendrocytes or OPC, which detect cells specialized for myelin production or precursor species that can generate myelin-producing cells, *then* investigating other functions of the selected cells. Examples of commonly used markers include OPC markers such as NG2 or PDGFR-α, myelin-related markers such as CNPase, neurite outgrowth inhibitor (NogoA), anti-adenomatous polyposis coli clone (CC1) or pan-OLG markers such as OLIG2.

Restricting analyses to cells committed to myelin-related functions is appropriate for investigating additional functions of these cells. Yet this overlooks the possibility that some cells assumed from morphology or location to be OLG do not have specialized myelin-related capabilities and instead may have other potentially even more important functions, such as iron regulation. We earlier reported most iron-rich glia with ‘OLG-like’ morphology (here meaning small size, eccentric nuclei and few processes) do not show myelin markers such as myelin basic protein or PLP in mice aged 12 weeks [57]. This appeared independent of location (juxta-myelin, juxta-neuronal, juxta-vascular or interstitial).

Ferriglia are common and widely distributed in healthy and pathogenic mouse CNS, being present in cortex and deep gray matter as well as white matter. Yet they are unlikely to have been detected in many past studies with markers such as those above, specific to cells committed to myelin formation or their precursors, specifically OPC, COP or oligodendrocytes. In healthy mouse [76] or human brain [68,69,106,107], their small size may cause them to be overlooked and their oval or ‘raindrop’ shape, eccentric nuclei and few fine processes of variable length/branching (Figure 2) may cause them to be mistaken for OLG or other glia. Ferriglial iron levels in brains with healthy iron balance can also be near or below sensitivity thresholds of histochemical stains, especially in leached post-mortem tissue.

We propose these cells are not members of known OLG or other glial compartments. It is possible that OLG and Fe+glia lineages arose by divergence from a common early progenitor cell. Alternatively, self-renewing mitotic Fe+glia may reside upstream of known OLG lineage compartments and, possibly in response to myelination-related signals, may generate downstream OLG progenitors by asymmetric divisions that also generate daughter Fe+glia. A third possibility is that there may be one or more separate ferriglial lineages that do not include OLG or other glia. More research into relationships between Fe+glia and OLG is required, including whether or not there are common evolutionary, lineage or developmental ancestors. Both current and emerging methodological approaches will be needed to confirm the existence of Fe+glia and understand their properties and origins (see also Section 4 and Section 6).

## 4. Rationales for the Separation of Iron Regulation from, and Precedence over, Myelination

Some, though not all, of the issues discussed above are recognized in attempts to define the nature of OLG by a core gene regulatory network, and the recognition that OLG may have had early primary functions in evolution other than myelination, e.g., [108]. Yet, as above, approaches focusing on regulatory genes identified in studies of cells with myelin-related functions or their precursor cells may not capture essential core regulatory features of cells with primary functions in iron regulation or other activities unrelated to myelin.

The high levels of iron in iron-rich Fe+glia assumed to be OLG and in myelin-containing structures are usually attributed teleologically to high requirements of myelination for iron (Section 4.2). As above, most Fe+glia in healthy brain are usually considered to be oligodendrocytes, other OLG, astrocytes or microglia. However we earlier showed that, in mice, most Fe+glia do not express the pan-OLG marker OLIG2 and, conversely, most OLIG2-expressing cells have little detectable iron, even in mice with high brain iron [57]. The morphologies of many Fe+glia, notably their small size, are also not consistent with those of oligodendrocytes or known oligodendrocyte precursors (Figure 2).

Below, we consider, in more depth, evidence for the possibility that at least some Fe+glia have primary roles in iron regulation rather than myelination.

### 4.1. Evolutionary Advantages of Separating Iron Regulation and Myelination

Early studies by Connor, Morris and others suggested some OLG might have functions in brain iron regulation and supply to other cells [68,71,74,75,76,107], but this has not been developed in the literature. It is widely, though often implicitly, assumed that most iron travels extracellularly through the brain bound to molecular carriers such as transferrin, ferritin or citrate, analogous to the blood. Instead, we propose some iron-rich cells with OLG morphology (Fe+glia) have primary iron-regulatory functions that include distribution of iron via their cell processes to other CNS cells, and not primary myelin-related functions. Consequently, whether these cells should be classified as OLG needs to be reconsidered.

Some juxta-myelin glia with high iron, ferritin or transferrin content might indeed be OLG, with primary roles in myelination or generation of myelinating cells; such cells may have subsidiary roles in iron transfer directly to the axon or soma of myelinated neurons by unknown mechanisms, perhaps via invagination of OLG membranes into the peri-axonal space [109]. Yet this appears unlikely to be the only cellular regulation of iron in the brain.

From a pragmatic perspective, there are likely to be strong evolutionary advantages of separating primary iron regulation and primary myelination functions. Having one cell type responsible for both may threaten survival in situations where these functions directly conflict. For example, myelinating cells could not relocate easily to take up iron as part of early neuroimmune responses at sites of hemolytic bleeding (Section 7.3) or pathogen invasion (Section 7.5). Similarly myelinating cells could not readily relocate to obtain iron from blood vessels or provide iron to cells in regions without nearby vessels or to myelin when required.

### 4.2. Rethinking Relationships of Iron, Myelin and Iron-Rich, OLG-like Ferriglia

Although high levels of iron in some cells with OLG-like morphologies, that are assumed to be oligodendrocytes or OPC, have been proposed to reflect high iron requirements for lipid and myelin formation, e.g., [107,110], there may also be other explanations [111]. Intense myelin formation during development or remyelination likely involves high iron usage. Yet it is unclear why myelinating oligodendrocytes in healthy adult brain, with slow myelin turnover [112], would require more iron than neurons, which usually have higher mitochondrial and metabolic activity than myelinating oligodendrocytes in healthy brain [113], yet lower iron levels than other brain cells [57,114].

We have suggested hydrophobic, lipid-rich, myelinated or myelin-like structures may provide safe reservoirs for brain iron storage [57]. Ferriglia may deposit surplus iron in such structures (e.g., see Figure 2), with later retrieval and delivery to neurons and other brain cells not specialized for iron storage and vulnerable to iron toxicity. We propose myelinated or myelin-like structures and Fe+glia may have important primary functions in iron sequestration that confer strong survival advantages, independent of advantages from saltatory conduction of electrical signals, and that these primary functions preceded saltatory conduction in evolution (see Section 5.5).

This is not addressed effectively in the literature, which considers myelin-related functions the paramount definitive feature of cells classed as OLG. As above, many studies are intentionally designed, or unconsciously biased, to investigate only cells committed to myelination or generating myelination-competent progeny [38,75,77,83,115]. This circularity precludes testing the concepts proposed here, i.e., that some cells classified as OLG by morphology or location are primarily specialized for other functions such as iron regulation, not myelin-related functions, and that such cells may not reside in OLG lineage compartments.

Similarly, both histochemical [69] and single cell transcriptomic [116] studies often use markers for OLG such as the iron transporter protein transferrin, that may also detect Fe+glia, causing Fe+glia to be assigned OLG identities. Numbers of easily visualized, iron-rich Fe+glia are low in healthy brain, in the absence of iron dyshomeostasis, which may also cause these cells to be overlooked in such studies, e.g., within small groups of unassigned cells not labeling for markers used to assign different cell IDs.

Markers which specifically label Fe+glia are needed. However, histochemical staining for ferrous and ferric iron currently requires concentrated acid treatment (pH < 1) [44]. This destroys RNA, making marker identification by transcriptomic profiling challenging. Alternative experimental approaches are needed. Unsupervised clustering analyses of omics data may help identify cells with molecular profiles consistent with primary functions in iron loading or other iron regulatory roles (i.e., Fe+glia), possibly in conjunction with profiles consistent with proliferation competency. We hope this review inspires research initiatives directly examining this new paradigm, including innovative approaches to Fe+glia marker identification.

It is important to recognize that Fe+glia may exhibit relatively little upregulation of iron or proliferation in healthy brain with normal iron homeostasis. Differential comparative analyses of samples from models with normal versus elevated brain iron levels are likely to be required to address this. Here we instead consider indirect evidence from evolution research that nervous system cells specialized for iron regulation preceded cells specialized for myelin generation.

## 5. Evolutionary Emergence of Molecular and Cellular Mechanisms for Iron Regulation

**Figure 3:**
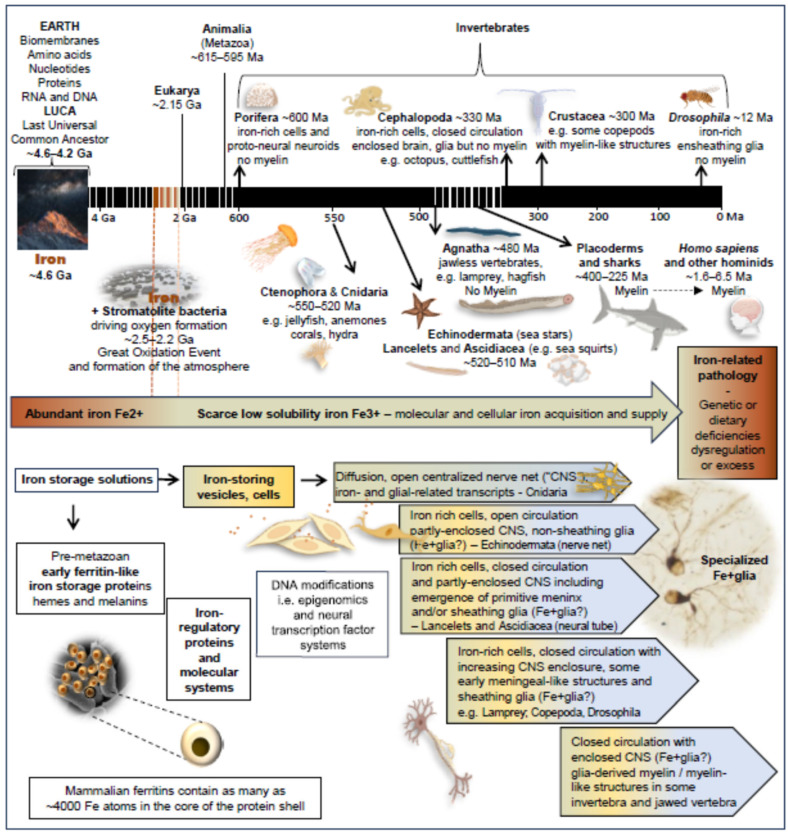
**The life story of iron.** Top timeline traverses events after solar system formation ~4.6 billion years ago (Ga) including: emergence of key biomolecular building blocks of life expedited by iron catalysis (lipids, proteins, RNA, DNA); last universal common ancestor (LUCA), a hypothetical single-celled organism with Fe-S cluster enzymes (Section 5.1.1); Great Oxidation event (Section 5); conjectured LCA at key evolutionary divergence points, e.g., emergence of multicellular Animalia and Bilateria, coinciding with emergence of early neural systems, and other important milestones on the road to the human brain (Section 5.1.2, Section 5.2 and Section 5.3). Time estimates are rounded to nearest 5 million years. Center timeline summarizes iron environments shaping terrestrial life, beginning with iron-rich environments with superabundant reactive ferrous iron (Fe^2+^) that kickstarted vital bioreactions in early single-celled lifeforms, including bacteria. Iron-poor environments increased after mass oxidation of Fe^2+^ to insoluble ferric ion (Fe^3+^), with evolution of diverse systems for effective iron acquisition and utilization in organisms already intrinsically dependent on iron (Section 5.1). Milestones in iron management solutions and nervous system development are represented lower down. These include ferritin homologs—prototypes of modern human iron storage systems enabling iron stockpiling, some already under iron response element control; membranous vesicles; specialized cells initially regulating iron at the whole organism level (‘ferricytes’; Section 5.1.1 and Section 5.2) and later regulating iron specifically within nervous systems (‘ferriglia’; e.g., Section 1), in conjunction with emergence of ensheathing glia and preceding the advent of myelin (Section 5.3). Time estimates are from [123], OneZoom Core Team (2025), OneZoom Tree of Life Explorer Version 4.1-59-g2fe26090 URL: http://www.onezoom.org [124,125] or as cited elsewhere in this paper. Images in figure are our own, public domain or icons created in BioRender.com.

Iron is the action hero in the story of life, a powerful catalytic enabler for a multitude of diverse bioactivities that have shaped all terrestrial organisms. Figure 3 gives a broad perspective of the story of iron and terrestrial life. Here we review evidence for iron as a primary driver in the emergence of life, independent of other early world views. These include Metabolism-First theories including hypotheses featuring iron, notably the coacervate theory [117], based on iron carbides, and Iron-Sulfur world [118], as well as Prions-First [119], Genes-First [120] and the popular RNA-First and RNA world views [121,122]. Irrespective of which world view prevails, we contend that iron is likely to have had central roles. In particular, there is strong evidence for our main theme—the emergence of iron-regulating systems and cells before the emergence of nervous systems and myelin.

Literature on evolution is continuously evolving. Aspects of the discussion below, such as dating, orders of emergence and properties of organisms and their iron-regulation and nervous systems, will in turn evolve as perspectives change, new information emerges and conflicting findings are resolved. Orders and timing of evolutionary events are based on recently revised reconciliation of molecular clock and fossil record estimates [123], OneZoom Tree of Life Explorer (https://www.onezoom.org/ accessed on 3 April 2025) [124,125] or other sources cited elsewhere, recognizing these estimates may alter as more information emerges.

### 5.1. Iron as a Powerful Driver of the Emergence and Evolution of Early Terrestrial Life

Iron was a prominent constituent of the early planetesimals that formed after the Sun was created ~4.6 billion years ago (Ga). It was abundant in the inner core of the newborn Earth, which formed ~4.5 Ga, and subsequently dispersed to the outer layers via convection currents, volcanic activity and hydrothermal vents [126,127,128,129,130,131]. Below we describe some of the ways this early abundance, together with the unique properties of iron, have shaped life on Earth.

#### 5.1.1. Iron and the Emergence of Early Macromolecules and Membranes

Iron is an avid electron trader. It can exchange electrons with hydrogen, sulfate and other important early elements and has been cast as the primeval driver of early biochemistry [130,132,133,134]. It can exchange electrons with oxygen and non-heme iron at oxygen-binding sites is thought to have been involved in oxygen-generating photosynthesis by stromatolite cyanobacteria that drove early oxygen formation (~2.5 Ga), culminating in the Great Oxidation Event (Figure 3) that formed the oxygenated atmosphere [131,135,136]. From prevailing geochemical and biochemical perspectives, iron catalysis and iron-containing compounds with catalytic capabilities, such as iron-sulfur (Fe-S) clusters, likely preceded emergence of larger molecules. Iron can catalyze generation of amino acids [137], peptides [138] and nucleobases [139] under conditions similar to those likely to have existed in early sediments and hydrothermal vents [140]. Iron is also able to catalyze simple bilayer membrane formation. Sequestration of iron in phospholipid bilayers, vesicles, endomembranes or other organelles and, ultimately, specialized cells can help drive biochemical reactions by creating high local concentrations of iron, providing safe storage reservoirs for iron and protecting against iron theft by competing life forms [126,127,141,142,143,144,145].

It is hypothesized [146] that enzymes containing Fe-S clusters were already present 4 billion years ago in the Last Universal Common Ancestor of all terrestrial lifeforms (LUCA; Figure 3), a primitive prokaryote [147]. Iron continues to have essential roles in vital bioprocesses in almost all known cellular species from Bacteria, Archaea, Eukaryotes and plants through to Animalia, with diverse functions spanning generation of energy, synthesis of proteins, RNA, DNA and other macromolecules, oxygen respiration and cell replication, amongst others.

As detailed below, we hypothesize that nervous system glial cells specialized for iron regulation and storage, i.e., ferriglia, originally evolved from cells with primary functions in iron-regulation at the whole organism level—i.e., ‘ferricytes’—that were already present in multicellular animals before the emergence of the nervous system. We further hypothesize that the iron-regulating cells in animals in turn arose initially from iron-regulating, non-animal ancestral cells.

#### 5.1.2. Iron in Non-Animal Organisms

Iron-regulating systems were central to the existence of essentially all terrestrial lifeforms for over three billion years before multicellular Animalia and the simplest nervous systems arose. This in turn was over a hundred thousand years before myelination emerged (Figure 3). Early organisms evolved many systems for acquiring, using and storing iron [148]. Complex regulatory systems featuring homologs of most of the proteins central to human iron regulation are present in bacteria and other uni- and multi-cellular non-animal organisms such as fungi and plants [149,150,151,152]. Examples include proteins resembling human proteins such as the iron storage protein ferritin [153,154], iron regulatory protein 1, frataxin (FXN), which can serve as an iron sensor, ferroportin (solute carrier SLC40A1), Nramp 1 (SLC11A1), divalent metal transport DMT1 (SLC11A2, Nramp2) and ferric chelate reductases [155,156]. For example, phytoplankton have highly sophisticated machinery for acquisition of iron, the main factor limiting their reproduction [157].

### 5.2. Iron and the Evolution of the Nervous System in Animals (Animalia)

The ubiquity and complexity of iron-regulating molecules in early animals such as sponges, jellyfish, corals and placozoa testify that vital iron regulatory systems important for unicellular and multicellular non-animal life were present in the hypothetical last common ancestor of all animals (Metazoa), a multicellular organism living over ~600 million years ago (Ma) [123,158,159].

Order of emergence of early animals such as sponges (Porifera), comb jellyfish (Ctenophora) and other early phyla (e.g., Placozoa, Cnidaria) is contentious [123,160,161], in part reflecting parallax of data from different sources e.g., fossil records, genomics [162,163]. It is also hard to map nervous system evolution accurately, partly due to lack of consensus on what constitutes a nervous system, a question beyond the scope of this review that is debated elsewhere [164,165,166].

Nervous system evolution is often framed as proceeding from simple nerve nets via segmentation, ganglia and spinal cord formation to the complex brain. To explore glial evolution in particular, from a functional perspective, we instead define stages in central nervous system evolution based on increasing integration and enclosure: i. proto-neural cells, with some features of both early neurons and early glia, ii. nerve networks (proto-nervous systems) comprising communicating nets of neural cells without clear integrated control, iii. integrated (‘centralized’), unenclosed nervous systems, including nerve rings, iv. integrated, partially enclosed nervous systems without meninges and v. integrated nervous systems fully enclosed in meninges or meninx-like structures [167,168,169,170,171].

Here we cover Porifera and Placozoa, without neurons or nervous systems [172,173], before Cnidaria and Ctenophora, with diffuse neural nets and sometimes even centralized nervous systems (Section 5.2.2). While order of emergence of these phyla is debated, this accords with recent reconciliation of fossil records, molecular clocks and other analyses putting Porifera first [123,161]. Regardless of order, the key point here is that molecules and cells with specialized iron-related functions are present in all these early phyla.

#### 5.2.1. Iron in Sponges and Other Early Animals Without Neurons or Nervous Systems

Sponges, among the earliest metazoa, contain transcript or protein homologs of various important human iron-regulatory molecules, e.g., ferritin heavy chain (FTH; confirmed at the protein level [174]), the iron carrier transferrin, ferroportin, divalent metal transporter-1 (DMT1 [174]), iron-regulatory protein 1 and heme synthesis components [173,174]. Iron-responsive, self-renewing, pluripotent stem-like cells may also be present [175,176,177]. Though usually considered to lack neurons, some sponges possess proto-neural forerunners of nervous system cells [175,177], termed ‘neuroids’ [178], and early forms of iron-dependent human neurotransmitters, e.g., GABA and glutamate [179,180,181,182].

Specialized iron-regulating ferricytes may also be present in another very early phylum, Placozoa, comprising small, disk-shaped marine creatures with few specialized cell types. Placozoa can preferentially acquire iron in gastrodermal cells by uptake of ferritin [183], suggesting specialized functions in iron handling, and some placozoa express the iron transporter melanotransferrin or the non-response to iron deficiency 1 (NRF1) protein, a transferrin homolog coordinating iron balance [184,185,186]. Although no neurons or glia have been identified, a few specialized cells expressing neural transcription factors such as NEUROD and OLIGs 1–3 may be proto-neural forerunners of nervous system cells [186].

It is unknown how early proto-neural or proto-neuronal precursors evolved to neurons, defined here as electrically excitable cells with specialized projections having primary functions in secretory or electrical signaling (adapted from [187]). Neurons and support cells (‘glia’) probably arose more than once in evolution and may have devolved in some phylogenetic lineages, contributing to uncertainty of phyla emergence order (Section 5.2). Greater understanding of iron regulation may come from detailed lineage characterization in early species, as underway for Placazoa, with neural and iron-related molecular profiling [186].

#### 5.2.2. Iron Regulation in Jellyfish and Other Animals with Primitive Nervous Systems

Like placozoans, ctenophores (comb jellyfish) can take up and digest ferritin in specialized cells [188]. Some cnidarians (e.g., other jellyfish, sea anemones, corals, hydra) also have complex iron-regulatory systems, including proteins such as ferritins [189], ferrochelatase and FXN, important in Fe-S complex synthesis for mitochondrial energy generation [190,191,192]. In humans, FXN mutations can cause neurosensory or motor impairment (Section 7.1.2), suggesting FXN may have provided Fe-S clusters essential for mitochondrial activity in early neuronal prototypes.

Nervous systems are present in extant members of these early emerging phyla, being made up of neurons connected by networks of inter-communicating cell processes that form diffuse nets, partially condensed plexuses or nerve rings [165,193,194]. Key iron-dependent neurotransmitters are again observed (e.g., glutamate, GABA, dopamine [193,195,196,197]), suggesting iron was already central to many activities in the earliest nervous systems.

Based on glia-related genetic profiles [198,199], glia have been proposed to be present in these early nervous systems, in particular in the cnidarian sub-phylum Anthozoa (e.g., coral, sea anemones, fans) [199]. However glia do not seem to have been identified in cellular analyses of representative model species, including the anemone *Nematostella vectensis* which instead has neural cells that have some glial properties i.e., proto-glia (reviewed [199]). Consequently glia are not usually considered to be present in these early nervous systems and are thought to have evolved later in Bilateria (bilaterally symmetric organisms) [193,195,196,197,199].

In contrast, the CNS appears to have emerged earlier, in or before the last common ancestor of Bilateria, e.g., [200], thought to have existed ~570 Ma [124,125], suggesting glia may not have emerged in parallel with centralization. Notably, although organisms in Ctenophora and Cnidaria typically have radial symmetry and are not classed as Bilateria, nerve rings in these phyla can constitute a single, integrated, ‘centralized’ command center for high level control of functions of ‘peripheral’ neurons in tentacles or ‘arms’, i.e., a central nervous system [165,193,194]. These deceptively simple systems can perform neurosensory and motor control functions, overriding local nerve control of individual arms for coordinated functions such as predation or jet propulsion and for complex behaviors such as navigating by sun compass or visual cues and using individual tentacles to deliver captured prey to the mouth [196,197,201,202].

‘Division of labor’ between ‘proto-glia’ and ‘proto-neurons’ probably began in early animalian phyla. Substrates and enzymes for synthesizing glutamate and other neurotransmitters are restricted to non-neuronal cells in some cnidaria [193,195,196,197,199,203,204]. Electrically active epithelial cells ensheathing neuron bundles in jellyfish and other early species are also considered to provide dedicated neuronal support that can be deemed to be a ‘glial’ function [165,198,205].

Separation of iron-regulation from information handling is likely to confer strong evolutionary advantages, due to high toxicity risks of storing iron in cells specialized for signaling, where iron regulation is not the primary function. Ferricytes with iron-related functions that were not yet fully specialized for neuronal support and were still also serving other cell types may have been present very early in nervous system evolution in some species, throughout the emergence of proto-neurons and neurons. Such cells could be considered ’iron-rich protoglia’.

While this is not yet clearly demonstrated for the CNS, analogous phenomena occurred in the evolution of the eye, where iron is stored in melanin pigment that is often visualizable without staining. ‘Proto-eyes’ of larvae of the small cnidarian box jellyfish Tripedalia consist of a single pigmented photoreceptor cell expressing both opsin light receptive molecules and iron-containing dark melanin pigment that helps provide shadowing that can assist in determining light direction and protect against excessive light exposure [206,207,208]. Melanin can also provide safe storage for intracellular iron.

This ‘proto-eye’ is thought to have subsequently undergone asymmetric division into a specialized sensory neuron (photoreceptor) and a specialized, iron-regulating, pigmented glial cell providing metabolic support to the neuron [208], although this separation of function is likely to have evolved gradually over time. Such proto-eyes may have been forerunners of the human eye, with Dunaief and colleagues reporting iron-regulating genes involved in melanogenesis in vertebrate retina [209].

### 5.3. The Transition from Non-Myelinating to Myelinating Vertebrate Species

The examples above confirm that cellular iron regulation was present in very early stages of animal evolution and likely involved early iron-rich *proto-glial* cells. In this Section, we present evidence that evolutionary emergence of iron-rich *glial* cells substantially preceded the evolution of myelin. We fast-forward to much later in evolution and consider the complex iron regulatory systems, potentially including Fe+glia, which were present when myelin with key structural and functional features homologous to human myelin emerged. This coincided with the evolution of jawed vertebrates (rays and sharks) from jawless vertebrates (lamprey and hagfish). We then back-track from this well-established ‘endpoint’ to develop a new paradigm for understanding the factors driving preceding evolution from iron-rich proto-glia to iron-rich glia (i.e., Fe+glia) and the early ensheathment of neurons by ‘proto-myelin’ (Section 5.4.).

#### 5.3.1. Iron Regulation in Ensheathing but Non-Myelinating Jawless Vertebrates (Agnatha)

The switch from a non-myelinated nervous system without oligodendrocytes to myelin produced by oligodendrocytes is thought to have coincided with divergence of jawed (Gnathostomata) from jawless (Agnatha) vertebrates early in vertebrate evolution [47,210,211]. Only two known agnathan phyla still exist today, lampreys and hagfish. Lampreys, primitive ‘vampires of the deep’, have sucker-like mouths lined with rings of sharp teeth that latch onto prey and siphon out blood and flesh. Hagfish lack oral suction disks and have evolved other ways of feeding without a jaw [212], including burrowing within dead or dying prey and absorbing nutrients, including iron, through their skin and gut epithelia [213].

Research has focused on lampreys, which have evolved several complex mechanisms for regulating iron. These include unique iron metabolizing organs, specialized bipolar iron-loading cells, melanin-containing macrophages in regions with iron-related activities such as absorption or phagocytosis [214,215] and iron storage within adipocytes in fat tissue [216]. Pigmented cells likely to contain iron, occasionally with morphologies consistent with Fe+glia, appear around the periphery of the CNS by prolarval stage S15 [217]. Ferric iron in ferritin and small amounts of ferrous iron are present in round glycogen- and lipid-rich meningeal cells in larval and juvenile brain and spinal cord [218]. Lamprey homologs of key iron regulatory genes include transferrin, ferritin heavy chain 1, prohibitin 2, which may control ferritin synthesis, the iron response element/iron regulatory protein (IRE/IRP) system and iron-regulating bone morphogenetic proteins (BMPs) [215,219,220,221,222,223,224,225]. The BMP receptor neogenin is expressed in ependymo-radial glial cells [226], possibly analogous to mammalian tanycytes (Section 5.4.1).

The lamprey nervous system has some similarities to ours [227,228], with iron-dependent neuronal systems [227,229,230] and glial-related transcription factors and growth factors, e.g., *Sox10* ortholog *SoxE2*, *Nkx2.2*, which regulates OLG precursors, and *PDGFRα* [210,227]. These may indicate the presence of glial gene-regulatory systems in the vertebrate common ancestor [210,231]. While lacking myelin and myelinating cells, lamprey have ensheathing glia with some features of mammalian olfactory ensheathing cells (OEC; Section 3.3.5), combining axon wrapping with metabolic support [211,232]. Whether these glia have iron-regulatory roles, and their relationship, if any, to the iron-rich cells described above, is unknown.

#### 5.3.2. Evidence for Myelin in Fossils of Prehistoric Sharks—The Oldest Known Jawed Vertebrates

Mammalian myelin and myelinating glia arose relatively early in vertebrate evolution [233,234]. The earliest surviving jawed vertebrate species (gnathostomes) in which myelin resembling human myelin has been reported are cartilaginous fishes, including rays and sharks (chondrichthyes). Myelination is present in all chondrichthyes studied [47]. No known living species appear to bridge the gap between the non-myelinated jawless lampreys and well-myelinated jawed rays and sharks, so most research has focused on oligodendroglia and myelin in vertebrates, beginning with sharks and other cartilaginous fish and moving forward.

It is proposed the myelin sheath and the hinged jaw were acquired ‘together’ in evolution, with both arising from the neural crest, which gives rise to the jaw and most of the peripheral nervous system [47,235]. ‘Together’ in this context should perhaps be interpreted loosely as meaning within the same extended time window, without the respective evolutionary pathways necessarily being co-dependent or temporally coincident. In any event, this appears to be well after the emergence of glia, including the iron-rich glia described for lamprey above. Zalc and colleagues report evidence for myelinated axons in a fossilized placoderm from the Devonian period (~420–360 mA), the oldest jawed fish known, and propose this is consistent with myelin similar to our own first emerging at this time [235].

Yet, as will be discussed below, different types of glial support cells [108,236,237] and myelin-like substances [238,239,240] arose independently multiple times in evolution, in invertebrates as well as vertebrates. Consideration of these other forms of myelin-related substances provides additional insights into myelinating glia and other ensheathing glia and the functions of the substances they produce.

### 5.4. Nervous System Enclosure and the Emergence of Ferriglia and Ensheathing Glia

Glial support cells that coat portions of neuronal bodies or axons with non-myelin, proto-lipidaceous substances are present in various species closely related to vertebrates within the bilaterian superphylum Deuterostomia. This superphylum eventually gave rise to *Homo sapiens* and includes the major clade Chordata of which vertebrates are a subphylum. Myelin-like substances are also present in some species in the other main bilaterian superphylum Protostomia, which diverged from Deuterostomia ~570 to 560 Ma, and gave rise to arthropods, insects and many other species. Glia are proposed to have been present in the last common ancestor of Protostomia and Deuterostomia [241] and, if this is correct, are also expected to be found in all descendant species except when lost through devolution.

Here we will first discuss non-myelinating ensheathing glia in early deuterostomes, with dorsal, primarily tube-like centralized nervous systems and peripheral ganglia, before moving on to protostomes, with ventral, primarily ganglionic, nervous systems (Section 5.5). As noted above (Section 5.2.2), glial evolution is usually considered to relate to nervous system ‘centralization’. We instead hypothesize that factors limiting direct access of nervous systems to external nutrients or restricting nervous system regeneration were the main evolutionary drivers of the emergence of glial cells with dedicated functions in the metabolic nurture or physical protection of neurons.

These limiting factors are likely to have included increases in organism and nervous system size, complexity or enclosure, and transitions to non-marine environments. Such constraints can vary considerably even between closely related species and, as further discussed below, may provide more plausible explanations for the diverse evolutionary pathways by which glia may have emerged (and may sometimes later have been lost) than nervous system centralization per se.

Regardless of the emergence pathways involved, storing surplus iron and supplying iron to neurons or other brain cells were probably early functions of glia, which are more expendable and easily replaced than post-mitotic neurons residing in complex, established communication networks, and hence better suited for storing essential but potentially toxic substances such as iron.

Glial cells with primary iron-regulating functions (Fe+glia) may have arisen by repurposing of existing generic iron-regulatory cells that eventually became sequestered with neurons from the rest of the organism, not by de novo development of iron trafficking by glial cells already solely serving the nervous system, as is sometimes suggested (e.g., [100]).

**We propose glial support roles co-evolved with segregation of nervous systems from the rest of the organism by brain barriers restricting exposure to the blood, cerebrospinal fluid, ‘glymphatics’ or other circulatory systems and by enclosure within protective coverings such as cartilaginous capsules or meninges.**

#### 5.4.1. Brain Barrier Systems

The barriers between the human brain and the rest of the body mainly consist of specialized endothelia (blood-brain barrier) or epithelia (choroid plexus; arachnoid epithelia). However in early organisms, prototypal barriers separating nervous systems from the rest of the organisms were predominantly glial [242,243,244,245]. This is exemplified by some members of the early Bilateria subphylum Echinodermata, deuterostomes with water vascular systems arising ~509 Ma such as sea stars, urchins and sea cucumbers. These organisms have nerve rings with radially arranged nerve cords, similar to Cnidaria and Ctenophora. In some echinoderms, nervous systems are partly segregated from the rest of the organism by a barrier of non-ensheathing glia lining the epineural canal, a forerunner of the cerebral ventricles in the human brain [246,247,248].

Modern analogs of these glia in humans and other vertebrates include tanycytes, which in rat brain stain strongly for iron and ferritin, as well as other ependymoglial cells contributing to brain barrier formation in the cerebroventricular lining [68,237,249]. Research is needed to determine if analogous iron-rich glia that may have specialized functions in regulating iron supply to centralized nervous systems are present in echinoderms. If so, Fe+glia are likely to have been present in nervous systems of multicellular organisms over 100 Ma before OLG cells with primary functions in myelination arose in early jawed vertebrates.

Evidence for glial-like neuronal support cells in early evolution is not restricted to Echinodermata. For example, other early bilaterians with glia-like cells supporting neurons in simple nets also include Xenacoelomorpha, early invertebrate worms arising ~540 Ma, where glia-like cells may have evolved convergently at least five times [250]. Again there appears to be little information on iron-regulating cells in these species.

#### 5.4.2. Brain Enclosure by Meninges and Related Structures

There is also little information on the evolution of meninges-like structures enclosing the brain. The two chordate deuterostome subgroups preceding Vertebrata are the Lancelets (cephalochordates, also called amphioxus), small eel-like animals which emerged within ~10 million years of Echinodermata (Figure 3) and the Tunicata (urochordates), which include Ascidiacea, sea squirts that have a tadpole-like larval form. In ~1899–1902, Italian anatomist Giuseppe Sterzi reported a spinal meninx comprising a single mesenchymal leaflet in Lancelets and its evolution in later species to the external dura mater and an internal ‘secondary meninx leaflet’ which then gave rise to the arachnoid and pia mater [251], but there has been almost no research in this area since then.

The simple Lancelet frontal eye at the tip of the cerebral vesicle (homolog to the vertebrate brain) has approximately six photoreceptor cells, nine pigment cells (homolog of retinal pigment epithelium) and three rows of putative neuronal homologs; the pigment cells appear capable of iron-regulation, expressing MITF and melanin synthesis genes, as reviewed elsewhere [252]. The Lancelets are also the earliest phylum known to have primitive meninges and are an early prototype of CNS enclosure, with a primitive neural tube and rudimentary ‘brain’. Perhaps not coincidentally, Lancelets are also the earliest known examples of deuterostomes exhibiting glial ensheathment of neurons, though ‘true’ multilayered myelin is not present [253,254,255,256].

We hypothesize proteolipidaceous glial-derived sheathing provides Lancelet neurons with readily accessible stores of lipids and, importantly, iron for essential functions such as energy generation, along with other nutrients such as fat-soluble vitamins. We conjecture this is an early prototype of mechanisms for the provision of nutrients or energy (via lipid metabolism) to axons from oligodendroglia and myelin in the mammalian brain [38,198,257,258,259,260,261]. Meninges protect nervous systems against pathogens competing for host iron and other nutrients, as well as from physical and chemical trauma. The meninx may have co-evolved with local storage of lipids, iron and other nutrients within nervous systems. Whether meninx evolution initially drove or was secondary to accumulation of internal CNS nutrient stores is unknown, but internal stores were likely present before maximal CNS enclosure by meninges.

There is evidence for cells involved in iron regulation within, as well as outside, the nervous system and for non-sheathing glial cells in the urochordate deuterostomes Ascidiacea, sea squirts with primitive neural tubes that evolved within ~10 million years of Echinodermata (Figure 3). The CNS of the tadpole-like ascidian larva also has neurons and glia, with two large pigmented, non-neuronal sensory cells containing melanin [262,263,264]. This may be synthesized at greater rates when dietary iron is abundant [265], consistent with regulated iron storage functions. Iron does not appear to have been investigated in ascidian neural cells using sensitive, enhanced histochemical iron detection methods such as those described in Section 6.

As noted above (Section 5.3.1), ferric and ferrous iron are present in meningeal cells in lamprey larvae and juveniles [218], consistent with the existence of Fe+glia in CNS barriers before the emergence of myelinating glia in early jawed vertebrates, e.g., sharks (Section 5.3).

It is beyond our scope to consider the many routes by which early nervous systems evolved or the uncertainty around orders and timing of emergence. This is often complicated by large differences between larval, juvenile and adult nervous systems even within a single species, as well as by difficulties distinguishing convergent evolution from devolution/loss scenarios and the possibility that sometimes less complex systems arising from ‘devolutionary’ loss of function may have evolutionary advantages in some environments.

While it is infeasible to trace precise evolutionary sequences, the examples above support the hypothesis that **specialized iron-regulating cells arose early in animal evolution and eventually gave rise to cells with roles in storing and transporting iron and probably also other nutrients within enclosed nervous systems**.

### 5.5. Evolution of Myelin-like Ensheathing Structures in Invertebrate Nervous Systems

We now turn to the protostome invertebrates, with ventral, mostly ganglionic nervous systems, and examine the emergence and persistence of proteolipidaceous, myelin-like ensheathing structures around neuronal soma and axons. In evolutionary contexts, ‘true myelin’ usually refers to compact human or vertebrate myelin conferring biologically significant increases in axonal conduction rates. This is generally deemed superior to invertebrate myelin and other proteolipidaceous structures that often (but not always) lack this property, despite similarities in composition, structure or other features.

However, although faster nerve conduction is widely considered the main driver for myelin evolution, this may be of less fundamental importance than is often assumed [72,73]. Concordant with the roles proposed for iron-regulatory glia above, we hypothesize that **the essential primary function originally served by proto-myelin and other neuronal ensheathing structures, and driving their evolution, is safe supply of iron and other ancillary nutrients to neurons,** and not saltatory conduction. We propose, in corollary, that saltatory conduction was instead a serendipitous downstream consequence of gaps arising from early incomplete ensheathment, that became a secondary supplemental evolutionary driver.

#### 5.5.1. Evolution of Invertebrate Myelin-like Structures

Like glia [108,237], myelin-like substances arose independently multiple times in protostome evolution [238,239,240]. In considering myelin evolution, it is important to recognize that while some researchers view ensheathing structures in the invertebrate nervous system as inferior to ‘true’ human or vertebrate myelin, some invertebrate ensheathing structures support very high conduction rates, comparable to vertebrates. Conversely, while the drivers for myelin evolution are usually considered to be the benefits of both energy savings and rapid nerve conduction conferred by saltatory conduction, especially in larger species, these may be less essential than often assumed [72,73].

For example, the phylum Cephalopoda, which arose ~330 Ma and includes octopus, cuttlefish and squid, contains some of the largest protostomes, with the largest brains. Glia are present and nerve fibers are sometimes thinly sheathed by myelin-like structures, yet cephalopods lack compact, conduction-enhancing myelin structures comparable to vertebrate myelin, with rapid conduction instead supported by large diameter axons [266,267,268]. The brains of many cephalopods are relatively complex and partly enclosed in a cartilaginous cranium, with a centralized component resembling the nerve rings mentioned earlier that control tentacular or other radial nerves in cnidarians, echinoderms and various other species, rather than vertebrate and other chordate brain structures [269,270,271]. Most nutrients are transferred from the cephalopod circulation (hemolymph) across a glial barrier [269,270,271] analogous to barriers described above for Echinodermata and below for Drosophila. This is again consistent with the possibility that local storage of iron and other nutrients in rudimentary ensheathing structures may have conferred early evolutionary advantages, rather than saltatory conduction, even in species with long axons.

Structures with increasing similarities to vertebrate myelin are present in various later arising invertebrates, e.g., some Crustacea such as crayfish and crabs [240,272]. Notably some small copepod zooplankton ~1–10mm long, arising ~300 Ma [268], have well-organized, multilayered myelin-like structures. While differing from vertebrate myelin in wrapping modes, insulation properties or substructures such as nodes of Ranvier, some of these structures, such as fenestrated shrimp ‘myelin’, support very rapid conduction rates [273], yet this does not necessarily translate to faster escape reactions [72,274]. Moreover many protostome myelin-like structures do not appear to confer any increase in conduction rate [72,73], again suggesting that the multiple instances of independent emergence and persistence of these structures reflect other, even more compelling, evolutionary advantages.

#### 5.5.2. Other Myelin-like, Lipid-Rich Structures That May Participate in Iron Storage

Intriguingly, electron microscopy suggests some myelin-like ‘ensheathing’ structures in these early species are not generated by glia and instead arise inside healthy neuronal axolemma or soma, sometimes though not always extruding out to contact other cells [238,275,276]. This phenomena is not restricted to invertebrates or to neural cells. Lipid structures resembling myelin by electron microscopy, termed myeloid, myelinoid or myelin figures or bodies, can arise in non-neural cells in various vertebrates, including cells in lamprey undergoing metamorphosis [277] and rat [278] or human hepatocytes or kidney cells [279]. Membranous whorls of laminated phospholipids are also observed within human HeLa cells exposed to iron or other amphiphilic cations [278,280], though this is usually only observed in pathological contexts, often with degeneration of lysosomes, endoplasmic reticulum or other membranous organelles.

As above, phospholipid bilayers can also form spontaneously in some conditions, and this may be accelerated by iron (Section 5.1.1), suggesting such structures may arise in response to high iron levels in the absence of specialized cellular synthesis machinery. Alternatively, some cells may retain ‘generic’ ancestral cellular machinery from early metazoan or pre-metazoan organisms for sequestering iron in vesicles or other membranous structures. Non-specialized cells may have only restricted capacity to store surplus iron safely, even by co-opting lysosomes or other membranous organelles, with excessive loading causing pathological organelle damage. Emergence of OLG-like cells primarily specialized for regulated generation of iron-sequestering membranous material could confer strong evolutionary advantages, although such cells may not themselves necessarily have initially had, or subsequently retained, primary specialized functions in iron storage or transport.

From anthropocentric perspectives, such structures may likewise sometimes be discounted as irrelevant to evolution of ‘true’ (i.e., human) myelin but we believe it is illuminating to recognize the existence of a spectrum of lipid-rich nervous system structures of differing origins and complexity that might confer other important benefits aside from insulation and accelerated signal conduction [274]. We propose the primary evolutionary advantage of such structures is their ability to function as safe ‘bulk storage’ reservoirs for iron and other nutrients, as well as for lipids themselves.

The evolution of advanced iron regulatory systems in invertebrates with non-myelin ensheathing structures is illustrated by Drosophila fruit flies, arising ~12 Ma. Drosophila brain is enclosed in a water-resistant protective cuticle formed predominantly of chitin, hydrocarbons and other lipids [281,282], with a blood-brain barrier comprised of glial cells [283]. Drosophila do not produce myelin but have ensheathing glia. Although proposed to resemble OLG more closely than other glia, despite not myelinating [284], these cells do not produce wrappings that contain Ranvier node equivalents [285] or appear to increase nerve conduction velocities [286,287,288,289].

Drosophila larval neural stem cells—‘neuroblasts’—appear to depend on exogenous iron provided by glia for survival, which is stored in ferritin, produced in all subtypes of Drosophila glia, including astrocyte-like glia and perineurial glia, as well as ensheathing glia [290]. Extracellular ferritin in Drosophila is high in iron, unlike serum ferritin in humans [291], and appears to be transferred from the glia to the neuroblasts in vesicles [290]. The Drosophila genome encodes multiple ferritin and transferrin homologs, which may be expressed differentially in healthy brain or in response to infection or other stressors [292].

Importantly, outside the nervous system, a Drosophila transferrin homolog transfers iron to fat bodies for sequestration during pathogen infections, as iron is less readily accessible within these structures [293]. It is feasible that iron is contained within ensheathing structures produced by Drosophila glia and that these provide a safe local reservoir of iron, lipids and other nutrients for neurons, although little research appears to have addressed this directly.

In sum, we have presented a new, iron-centric paradigm for understanding nervous system evolution that has generated testable, fundamental hypotheses about Fe+glia and also related hypotheses about the primary functions of iron-rich lipidaceous brain structures homologous to myelin that may not ensheathe axons. To test these concepts, further research is needed to clarify the roles and spatial relationships of iron-rich cells and lipidaceous structures—particularly in relation to neuronal nets, neural systems, barriers, and enclosing structures—across key evolutionary species in the development of nervous systems, iron-rich glia, myelin and other ensheathing substances. Species constituting important evolutionary milestones in this context include cnidarian, ctenophores, copepods, Drosophila, Lancelets, Agnatha and sharks. Below we consider techniques that may help overcome some of the problems which have limited past research in this area.

## 6. Technical Considerations

It is clear from the literature that there has been little appreciation of the fundamental importance of deeper understanding of the cellular regulation of iron in the human brain and other nervous systems across evolution. One big factor contributing to the blind spots in the literature and the lack of research in this area has been the technical difficulties studying iron, which can be redistributed or lost during tissue processing with organic solvents. Had Hortega had access to the more sensitive iron labeling techniques now available, it is possible that neuroscience and evolutionary research would have evolved very differently. Even today, few studies have used enhanced iron histochemical staining or other sensitive techniques to investigate the distribution of iron in nervous system cells and their ancestors in non-mammalian species. Below we briefly review historical and contemporary labeling or label-free methods for imaging iron at cellular and subcellular levels.

### 6.1. Challenges in Studying Iron and Other Metallic Elements in Brain Glial Cells

Iron is the most abundant transition metal in the human brain, but to gain full understanding of how iron in cells and tissues is compartmentalized and bound requires exceptional analytical sensitivity and specificity [294]. Viewing iron homeostasis as a system at the whole tissue level is key to determining its role in brain function in health and disease, and immunohistochemical analysis of proteins responsible for iron homeostasis can provide valuable insights [295]. Yet preserving iron chemistry in post-mortem tissue samples presents a key analytical challenge; conventional formalin fixation can incur substantive, unpredictable and compartment-specific effects on the levels of iron and other elements in tissue [296,297].

Conventional histological staining is also problematic for brain metals analysis. The Prussian or Berlin blue stain, developed in the 1860s by Max Perls and now commonly referred to as Perls’ stain [298], targets ferric iron but also detects ferrous iron, which is highly labile and rapidly converts to the ferric form [44]. Although Hortega trialed Prussian blue iron staining of human brain sections, he and colleagues achieved only pale staining of OLG by this method, perhaps explaining why he did not give due recognition to the possibility of OLG-like cells having iron regulatory roles. Many decades elapsed before this was revisited by James Connor, Torben Moos, ourselves and others [57,68,87,111,299,300], using modern techniques that included immunolabeling of iron-related proteins combined with enhanced Perls’ using DAB [44] or other agents.

Whilst suitable for conditions associated with iron over-accumulation, even with modern enhancements [44,87], Perls’ stain may sometimes lack the requisite sensitivity for representative visualization of normal iron levels in brain tissue [301]. In addition, the dyes used are engaged in a competitive exchange equilibrium with endogenous ligands and are therefore unsuitable for multi-metal analysis [301]. This is relevant when considering that metal transporters are commonly shared by different metals, so dysregulation of iron, for example, may cause knock-on effects for other essential metals and vice versa. Staining can also disrupt the native tissue chemistry and thereby limit accurate speciation of metal ions with regard to properties such as oxidation state [302]. For redox metals such as iron, this may be important in the context of oxidative damage mechanisms proposed to be among the factors implicating iron dysregulation in neurodegenerative disorders [295,303,304].

### 6.2. Small Molecule Fluorescence Probes and DNAzyme-Based Fluorescent Sensors of Labile Iron

New labeling approaches are now coming through that may reduce such problems and provide additional insights into brain iron homeostasis. In recent years, synthetic small-molecule probes have been developed that convert the binding of the small molecular target to fluorescent signals [305,306]. These fluorescent probes allow selective detection of Fe^2+^ that is loosely bound to surrounding molecules. Despite this advance, few such probes allow simultaneous imaging of metal ions in different oxidation states with high selectivity. The ability to monitor the same metal ion in different oxidation states is important to understand iron regulation in brain cells because iron has two oxidation states that may play different roles. For example, even if the labile iron is at the same level, different ratios of Fe^3+^/Fe^2+^ may have very different effects on lipid peroxidation [307,308], which is implicated in ferroptosis. This may play important roles in some neurodegenerative diseases such as Alzheimer’s disease (AD) [309], particularly in late disease stages.

To address this issue, the Lu group has undertaken the construction and initial characterization of highly selective DNAzyme-based fluorescent sensors for simultaneous imaging of each of Fe^2+^ and Fe^3+^ by using in vitro selection from a large DNA library of up to 10^15^ different sequences and converting to fluorescent sensors by the ‘catalytic beacon’ approach [310]. A DNAzyme-based sensor is a special type of biosensor that uses a DNA molecule with enzyme-like activity to detect specific substances. These sensors work by combining a DNAzyme with a detection system, such as a color change or fluorescence, to signal the presence of a target, such as metal ions, small molecules or genetic material.

As described elsewhere [310], iron sensing DNAzymes utilize RNA-cleaving DNAzyme, which consists of an enzyme strand and a complementary substrate strand containing a single RNA linkage at the cleavage site. A fluorophore is conjugated to the enzyme strand, while two quenchers are positioned at opposite termini of the enzyme and substrate strands to suppress fluorescence in the intact state. Upon Fe ion binding, the DNAzyme catalyzes substrate cleavage, lowering the melting temperature at which the two DNA strands separate. This separation physically distances the fluorophore from the quencher, resulting in a fluorescence turn-on signal proportional to ion concentration. Such DNAzyme-based Fe sensors offer a sensitive, selective platform that is a promising alternative to traditional sensors for detecting different Fe forms with high selectivity, specificity and tunability. Importantly, this design decouples the target recognition core from the signal output, which allows multiplexing by incorporating fluorophores with distinct emission wavelengths. This property allows simultaneous imaging of the same metal ions with different valence states.

Although Fe+glia were not described specifically in the initial report above [310], small iron-rich cells, some with visibly eccentric nuclei, consistent with features of Fe+glia described in this review, were picked up by the sensors in eleven-month-old 5xFAD mice and age, strain and sex-matched WT B6SJLF1/J mice (Figure 4A in [310]). The sensors may provide a valuable platform for studying labile iron species, as opposed to iron bound within protein structures. By differentiating iron states and distribution in brain tissue, DNAzyme-based sensors also have the potential to enhance understanding of iron valence states in Fe+glia and other cells under different conditions and their contributions to iron homeostasis in health and pathogenesis, offering additional insights that may inform targeted therapeutic strategies.

### 6.3. Label-Free Imaging of Iron and Other Metallic Elements

Nowadays, the capacity to generate image contrast in a label-free manner using increasingly sophisticated chemical imaging techniques such as X-ray spectromicroscopy can also provide important alternative perspectives. Depending on the particular method applied, this class of techniques offers potential for multi-metal analysis of tissue samples at micro/nanometre length scales, with outstanding sensitivity and specificity [311], the option of collecting complementary information on metal chemical state and the potential to map, in the same experiment, corresponding distributions of certain organic components such as neuromelanin or amyloid beta [302,311,312,313,314].

Although modern multiomics is now being widely applied in evolutionary research, to test the ideas put forward here, multiomics needs to be performed in conjunction with established, as well as new, techniques for analyzing iron. This will be pivotal in advancing knowledge of nervous system iron regulation at the cellular level and understanding the neurobiology of iron. It will also be important for gaining insights into the roles of Fe+glia in nervous system disease.

## 7. Involvement of Iron-Rich Cells Resembling OLG in Neurological Conditions

Iron and OLG-like cells that may include Fe+glia are widely studied in myelin disorders. Yet if Fe+glia help maintain healthy iron balance of neurons and other brain cells, Fe+glia dysfunction is likely to be implicated in iron imbalances in other brain conditions not primarily involving myelin. The roles of iron in fundamental brain processes likely to be important for mental health are too numerous to address here, however we have briefly reviewed some of the neuropsychiatric conditions involving iron imbalance elsewhere [57]. Below we consider various other brain conditions likely to involve Fe+glia.

### 7.1. Iron-Rich Glia in Brain Diseases Involving Mutations in Iron-Related Proteins

Pathology of iron-rich brain cells with OLG-like morphologies—putative Fe+glia—is seen in some conditions involving mutations in proteins with primary or secondary roles in iron homeostasis. Two examples are discussed below.

#### 7.1.1. Hereditary Neuroferritinopathy

Rare mutations in ferritin light chain 1 (FTL1), important in iron storage, cause hereditary neuroferritinopathy, a member of the ‘neurodegeneration with brain iron accumulation family’ (NBIA) [315,316,317,318]. It usually presents with chorea, dystonia or, less commonly, parkinsonism, progressing to severe motor disability and speech and swallowing deficits. It features cytoplasmic and nuclear ferritin inclusion bodies and increased levels of intracellular labile iron, with coalescence of abnormal intracellular iron and ferritin aggregates [319,320,321]. There is patent pathology and sometimes death of OLG-like cells, including small through to large intracellular iron or ferritin aggregates that sometimes almost fill the cell body, with astroglia and neurons also affected, and extensive cell death throughout grey matter as well as white matter and myelin loss [319,320,321,322]. It appears likely that Fe+glia are affected, as well as recognized glial species, but the nature of Fe+glial involvement remains to be determined.

#### 7.1.2. Friedreich’s Ataxia

Friedreich’s ataxia (FRDA) is a rare genetic disease with characteristic symptoms of progressive cerebellar and sensory ataxia and limb dysmetria, primarily involving neuronal atrophy and synaptic degeneration, with myelin pathology considered secondary [323]. It is caused by reduced frataxin expression and function due to *FXN* gene mutations [324]. Frataxin regulates synthesis of mitochondrial Fe-S clusters (see Section 5.1.1) needed to make ATP [325] and is highly conserved in most prokaryotic and eukaryotic cells [326,327] (see also Section 5.1.2). While not known to regulate iron directly, it may do so indirectly by altering heme synthesis through perturbation of mitochondrial respiratory chain function or other mechanisms.

Iron accumulation and related pathology only occur in specific nervous system regions in FRDA [325], consistent with heterogeneous mechanisms of iron regulation. The cerebellar dentate nucleus exhibits neuronal atrophy and synaptic terminal abnormalities that may reflect mitochondrial iron dysmetabolism [328]. Re-distribution of iron or ferritin from cells morphologically resembling OLG to other glia [328] could reflect astrocytes or microglia subsuming iron-regulatory functions or phagocytosing iron-containing materials due to dysfunction or death of specialized iron-regulating cells (Fe+glia). Effective treatment of FRDA may need to address the heterogeneous nature of iron homeostasis in different nervous system regions and cellular elements.

### 7.2. Iron-Rich Glia in Brain Diseases Involving Amyloids

Pathology that may involve Fe+glia is also seen in various common or rare brain diseases involving amyloid, including AD, certain α synucleinopathies and transthyretin (TTR)-related siderosis.

#### 7.2.1. Alzheimer’s Disease

Iron-rich cells with OLG-like morphology have been observed in AD, which features cognitive impairment and deposition of plaque structures containing Aβ amyloid aggregates. As in normal human brain, iron in post-mortem AD brain is observed in cells with OLG-like morphology, with eccentric nuclei positioned towards one side of the cell—i.e., features consistent with Fe+glia. Transferrin or ferritin, individually or together, have been reported in these cells in normal brain and AD brain [68,69,106] and similar iron-rich cells with OLG-like morphology are often seen surrounding plaque structures containing Aβ amyloid aggregates in post-mortem brain from AD patients [76,106,329].

While literature on OLG-like cells in AD largely focuses on myelin and white matter loss [330,331], interactions of Aβ with Fe+glia might interfere with important functions of Fe+glia not involving myelin, such as iron supply to neurons or other brain cells or clearance of excess iron. This is also relevant to other conditions involving Aβ or other forms of amyloid. Notably, abnormal brain iron deposition (siderosis) has attracted recent attention in the context of severe, sometimes fatal responses to new immunotherapies targeting the Aβ peptide, sometimes also involving cerebrovascular amyloid angiopathy [332,333,334]. Understanding the biology of iron-rich glia may provide unexpected insights into these phenomena.

#### 7.2.2. Superficial Siderosis with Amyloidosis

Superficial siderosis, featuring build-up of hemosiderin in the subarachnoid space of the brain or spinal cord, usually arises from iron deposition secondary to cerebrovascular hemorrhage. However it can also be caused by other factors, including co-aggregation of iron and amyloid, as illustrated by meningovascular amyloidosis of the thyroid hormone distributer TTR [335]. Amyloid derived from TTR differs in protein composition from Aβ amyloid but can still cause abnormal glial iron accumulation. This is illustrated by siderosis, superficial cortical reactive gliosis and abnormal cortical glial iron accumulation in the autopsy of a patient with dementia, ataxia and leptomeningovascular, subpial and subependymal TTR amyloidosis, with little evidence of microvascular hemorrhage or myelin pallor [336]. One of us (SR) has shown pluripotential stem cell fate may be shifted towards OLG myelinating phenotypes in TTR null mice [337,338,339,340], with increased ‘oligodendrogenic to neurogenic’ ratios [341]. This suggests TTR normally limits cell fate commitment to the OLG lineage. As glial iron homeostasis can be disrupted in patients with some TTR mutations, as above, it is possible TTR has direct or indirect roles in brain iron regulation involving Fe+glia that may affect normal brain functioning.

#### 7.2.3. α-Synucleinopathies

The α-synucleinopathies are characterized by intracellular protein inclusions in neurons (e.g., Parkinson’s disease; PD), or glia (e.g., multiple system atrophy; MSA). While OLG changes can occur in PD or related diseases such as progressive supranuclear palsy that sometimes exhibit synuclein abnormalities [342,343], research on synucleinopathy of OLG lineage cells has mainly focused on MSA, a severe, fast-progressing, adult onset degenerative disease featuring impairment of the autonomic nervous system and movement [344]. The hallmark of MSA is cytoplasmic inclusions containing α-synuclein in glia usually considered to be OLG, correlating with measures of pathogenesis [345]. Iron homeostasis may be dysregulated, as evidenced in MSA post-mortem brain [346], with regional or cellular redistribution, e.g., accumulation at some sites and functional deficiency at others [295,347]. It is unknown if Fe+glia have counterparts among iron-rich cells in MSA brain (e.g., [295]), if dysregulated glial iron homeostasis affects OLG maturation in MSA or, conversely, if perturbation of differentiation or maturation of OLG lineage cells and/or Fe+glia drives iron dyshomeostasis. It is also unknown how, if at all, any of these phenomena relate to α-synuclein aggregation.

OLG lineage specification and function appear dependent on tightly regulated gene expression changes determined by DNA methylation and other epigenetic mechanisms [348]. Epigenetic studies confirm dysregulation of OLG-related genes in MSA e.g., the *MOBP* gene encoding myelin-associated oligodendrocyte basic protein [346,349,350]. It is unknown if decreased levels of *MOBP* expression accompanying increased DNA methylation in MSA affect the differentiation capacity of OPC or, conversely, if decreased *MOBP* expression reflects a shift from OLG differentiation towards Fe+glia proliferation.

We saw decreases in transcripts for *MOBP* and other OLG-related genes in human post-mortem brain from two cases with neurodegeneration and brain iron accumulation without confirmed genetic etiology and in mouse models with elevated brain iron loading [351,352]. In mouse brains with elevated iron levels, iron remained mostly distributed between myelin and glia, with only a small number of Fe+glia co-labeling for the pan-OLG marker OLIG2 and few, if any, for the astrocyte marker GFAP or the microglia marker IBA1 [57]. It will be illuminating to elucidate the nature and directionality of relationships between alterations in both DNA methylation (or other epigenomic factors) and protein expression with proliferation or differentiation of Fe+glia or OPC. Relationships of OLG fate and dysfunction in MSA with α-synuclein glial cytoplasmic inclusions, iron dyshomeostasis and perturbation of iron-related proteins also warrant further investigation.

### 7.3. Cerebrovascular Disease and Stroke

Few studies have investigated OLG or glial iron in stroke. We propose that cells with OLG-like morphologies and functions in iron regulation in healthy brain might participate in iron management after stroke. This may put these cells at high risk of damage, in line with reports of pathology of OLG-like cells arising from hemorrhagic [308] or ischemic [353,354] insults. For example, in rats, cells presumed to be OLG in the infarct core, identified by morphology after hemotoxylin/eosin staining and electron microscopy, were observed to swell as early as 30 min post-stroke and die within 2 h [355]. While identities and functions of affected cells are rarely studied, this vulnerability could reflect high levels of iron, oxidative metabolic activity or glutamate receptor permeability, features consistent with non-myelin related primary functions.

Based on co-labeling for Ki67 and NG2 (OPC and pericyte marker), Sun and colleagues identified proliferation of OLG progenitors in the hyper-acute phase (<24 h) as a rapid compensatory response to ischemic stroke in rats, consistent with functions in salvaging penumbral tissue [356]. Changes may also occur at later stages. In a mouse model of ischemic stroke, Kishida and colleagues reported altered phenotypes and locations of OPC (identified by PDGFRα), from interstitial sites away from microvessels, to intermediate sites near microvessels or juxta-vascular sites, fully wrapped around microvessels [357]. It is unknown if affected cells in either setting (i.e., interstitial vs other sites) have high iron contents or other factors that might contribute to increased susceptibility to ischemic damage. Research is needed to assess involvement of non-myelinating cells with OLG-like morphologies and iron-regulating functions (i.e., candidate Fe+glia) in cerebrovascular disease and stroke responses.

### 7.4. Brain Cancers

Gliomas, the most prevalent and aggressive primary brain tumors, arise from malignant transformation of glial cells or their progenitors. Nutritional influences, including iron, feature in the multifaceted interactions of environmental risk factors with inherent genetic alterations that are proposed to modify glioma risk and progression from low-grade to high-grade gliomas [358,359]. Dysregulation of iron homeostatic systems is characteristic of gliomas, particularly glioblastomas, the most common and aggressive subtype of glioma, with aberrant expression of proteins such as the homeostatic iron regulatory protein HFE [360,361,362]. Iron is a promising target for glioma therapy due to its pivotal role in glioblastoma tumorigenesis. For example, long non-coding RNA (lncRNA) RP1-86C11.7 interacts with the microRNA hsa-miR-144-3p to suppress transferrin receptor 1 levels, thereby inhibiting malignant properties of glioblastoma cells, including their proliferation and survival [363].

Yet many current and emerging treatments fail to consider the complex cellularity of gliomas and surrounding tissue [360]. With specific regard to how proliferation-competent, iron-rich cells with OLG-like morphologies may contribute to gliomas, high iron uptake is observed in glioma stem cell-like cells compared to matched glioma non-stem cells [364,365,366]. The former are capable of self-renewal [360] and may share some features with Fe+glia observed in healthy brain. Targeting such cells by iron chelation or other strategies might be effective in reducing tumor proliferation. For example, iron chelators can induce cell death in glioblastoma cells in vitro [367,368]. However, such treatments might also impact normal iron-rich glial cells and, potentially, iron supply to neurons. Deeper understanding of proliferative Fe+glia is essential for developing safe, effective cancer therapeutics targeting brain iron.

### 7.5. CNS Infections and Neuroimmune Disorders

Some cells with OLG-like morphologies or expressing OLG-related markers in human brain or mouse models can manifest molecular profiles related to immune responses. Examples include expression of cytokine receptors or transcripts for genes involved in antigen processing and presentation, such as major histocompatibility complex (MHC) class I and II. This has been interpreted as showing that these cells can undergo phenotypic changes to express immune functions that can supplement or even take precedence over myelin-related functions and permit active participation in immune-related processes in healthy or disease-related contexts [23,93].

Whether OLG-like cells expressing immune functions include Fe+glia and the roles of Fe+glia in infection are unclear. Sequestration in cells or other membranous structures helps protect host iron from pathogens, as illustrated by iron-laden hemoglobin in red blood cells or containment of iron within other pigments such as hemerythrin or melanin (Section 5). As the main iron-sequestering cells in the healthy brain, Fe+glia are likely to be both frontline defenders against pathogens infiltrating brain tissue and, conversely, potential targets for iron theft by pathogens, yet iron is rarely studied in these contexts. As many as 4 in 5 people who acquire SARS-CoV-2 infections develop neurological symptoms that sometimes persist long-term [369,370] and new pathogens affecting the brain are likely to emerge in the future. Greater understanding of neuroimmune roles of iron-regulating glia is needed, irrespective of the eventual ontological classification of these cells.

## 8. Considerations for Iron Chelation and Other Therapies

Iron chelators have been trialed for some of the diseases above, specifically AD [371], PD [372], NBIA [373,374] and FRDA [375,376]. While some trials returned mixed or promising results (e.g., [375,376]), others were unsuccessful and sometimes even exacerbated disease, notably recent AD, PD and NBIA trials [371,372,373,374]. Patients with these conditions often do not display whole body iron overload, and may experience iron insufficiency with chelation [377]. Also, importantly, chelation effects may be very different for brain cells with different iron loading properties. For example, more aggressive chelation protocols might be needed to reduce iron in Fe+glia specialized for iron storage. However, conversely, aggressive chelation may directly cause iron deficiency in neurons, which are already low in iron, and could also indirectly interfere with iron supply to neurons and other cells by Fe+glia.

Developing selective therapeutic approaches for specific cellular targets to restore iron homeostasis in different disease contexts requires deeper understanding of the heterogeneous cellular elements of iron regulation, including Fe+glia. The astroglial, oligodendroglial and microglial lineages are still being refined and validated by numerous groups worldwide, despite extensive research over the past century. Conclusive confirmation and characterization of a new glial class is therefore likely to be a major undertaking, requiring research across a range of experimental systems and species, using diverse methodological approaches.

## 9. Conclusions

We have presented strong evidence across multiple fronts for a novel, fundamental neural cell group with primary functions relating to CNS iron regulation. Confirming the existence of these cells and understanding their properties and the implications for contemporary neuroscience, neurology and neuropsychiatry requires deeper knowledge of ferriglial subtypes in humans and other species, their evolution and where they reside in relation to recognized glial lineages and compartments.

If valid, the core ideas presented here will fundamentally reframe our understanding of the brain, its main cellular components, how iron has contributed to the emergence of life and the shaping of nervous systems throughout evolution, and how our brains control iron in health and disease. We opened with the importance of speculation for original observation and will close with acknowledgement of the importance of other functions that are also served by speculation [378]. While contemporary knowledge does not speak to the validity of all the ideas above, we have tried to use the fresh perspectives opened by our conjectures to interrogate and integrate existing knowledge in insightful and illuminating ways. We hope this informs ongoing debates, lays foundations for testing the hypotheses and stimulates new ideas-in this area, even if ours are wrong.

## Data Availability

No new data were generated or analyzed in support of this research.
